# Comparison of Nanomaterials with Other Unconventional Materials Used as Additives for Soil Improvement in the Context of Sustainable Development: A Review

**DOI:** 10.3390/nano11010015

**Published:** 2020-12-23

**Authors:** Gang Liu, Chong Zhang, Mingzhi Zhao, Wenbo Guo, Qiang Luo

**Affiliations:** 1 School of Civil Engineering, Architecture and Environment, Xihua University, Chengdu 610039, China; 0120130047@mail.xhu.edu.cn (G.L.); civilzhangchong@163.com (C.Z.); civilguowenbo@163.com (W.G.); 2 Institute of Geotechnical Engineering, Xihua University, Chengdu 610039, China; 3 MOE Key Laboratory of High-Speed Railway Engineering, Southwest Jiaotong University, Chengdu 610031, China; LQrock@home.swjtu.edu.cn

**Keywords:** sustainable development, nanomaterials, soil improvement, reinforcement mechanism, price/performance ratio

## Abstract

Since the concept of sustainable development enjoys popular support in the 21st century, various kinds of unconventional materials were introduced for soil improvement in the past few decades to replace the traditional materials like concrete and lime. This paper compared nanomaterials with other three kinds of representative unconventional materials to demonstrate its superiority in soil treatment. The other three kinds of unconventional materials include microbially induced calcite precipitation (MICP), recycled tire and environmental fiber. Nanomaterial and MICP have a comprehensive effect on soil reinforcement, since they can improve shear strength, adjust permeability, resist liquefaction and purify the environment. Recycled tire and environmental fibers are granular materials that are mostly adopted to reinforce reconstituted soil. The reinforcement mechanisms and effects of these four kinds of unconventional materials are discussed in detail, and their price/performance ratios are calculated to make an evaluation about their market application prospects. It can be seen that nanomaterials have promising prospects. Colloidal silica, bentonite and laponite present a satisfactory effect on liquefaction mitigation for sandy foundation, and carbon nanotube has an aptitude for unconfined compressive strength improvement. Among the investigated nanomaterials, colloidal silica is the closest to scale market application. Despite the advantages of nanomaterials adopted as additives for soil improvement, they are known for unwanted interactions with different biological objects at the cell level. Nevertheless, research on nanomaterials that are adopted for soil improvement are very promising and can intensify the relationship between sustainable development and geotechnical engineering through innovative techniques.

## 1. Introduction

Natural soils do not always have mechanical properties that are strong enough to meet requirements in civil engineering. In this case, a soil improvement technique is universally necessary in civil construction. In a broad sense, soil improvement mainly refers to the enhancement of shear strength and stiffness, reduction of settlement and lateral deformation, adjustment of hydraulic conductivity and mitigation of the risk of soil liquefaction [1,2]. Several kinds of soil improvement techniques are widely used in civil engineering, such as dynamic compaction, vibro-compaction, stone columns, underpinning, grouting and so on [3]. Although these methods can effectively improve the engineering properties of soil structures, notable limitations and problems still exist. For instance, the compaction method may cause noise and vibration to the surroundings during the construction process and is therefore generally not applicable in densely built areas. Moreover, as the most commonly used traditional modified material, cement slurry may be distributed unevenly in the treated ground when it is used as the grouting material for ground stabilization, and lime can just be adopted to treat reconstituted soil. Furthermore, chemical solutions may even pollute the underground water in the construction process. Therefore, traditional grouting materials can cause great concerns in terms of both engineering performances and environmental protection.

Since the sustainable development concept was proposed in 1984 by the United Nations World Commission on Environment and Development (UNWCED), the question of how to meet present needs without compromising the ability of future generations to meet their own needs is a major issue. In 1991, the International Union for Conservation of Nature (IUCN), the United Nations Environment Program (UNEP) and the World Wildlife Foundation (WWF) published “Protecting the Earth—Strategy for Sustainable Living” and defined sustainable development as improving the quality of human life without exceeding the carrying capacity of the ecosystem [4,5]. Since construction activities cause the largest consumption of energy and natural resources among human activities, civil engineering is considered as one of the most unsustainable practices in recent years. In this situation, reusability and recycling of waste materials have been gradually valued significantly. Moreover, the concept of “Green material” was also proposed to meet the performance requirements and support the concept of sustainable development without environmental damage. The new unconventional materials should be safe, convenient, economic and have environmental compatibility [5].

In the past 30 years, much research has been carried out on the materials used in civil engineering to meet the sustainable development concept. For example, bio-deposition and bio-mineralization can not only improve the concrete strength significantly but also reduce the mixed proportion of cement, leading to the reduction of CO_2_ emission in cement production process. In addition, a kind of volatile organic coating was used for high-strength concrete to inhibit crack propagation and reduce potential air pollution [6]. Except for the new technologies and materials that are adopted to improve the properties of concrete, recycled industrial wastes are widely used in geotechnical engineering. These industrial wastes are mainly some slag or rock debris like fly ash, bottom ash, blast-furnace slag, quarry fines and glass shards, etc., whose suitability has been confirmed as road or highway construction materials [7]. Moreover, research has also been carried out on sustainable organic materials such as bitumen, geotextiles, injection resins and composite materials. Their performances in soil improvement were greatly valued, and the service life of corresponding soil structures was carefully evaluated [8].

With the development of science and technology for emerging unconventional materials, nanomaterials and other three kinds of sustainable materials, i.e., microbially induced calcite precipitation (MICP), recycled waste tire rubber and environmental fibers, are extensively used in soil reinforcement. In this paper, these four unconventional materials are reviewed to demonstrate their significant effects on improving shear strength, inhibiting liquefication and adjusting permeability of foundation soil. For each method, the reinforcement mechanism is discussed and compared in detail. Then, improvement effects and characteristics of these four kinds of unconventional materials are evaluated. Finally, the cost performance of each unconventional material is estimated to make a simple judgment about their market application prospects in the near future. This work will provide guidance for the applications of unconventional materials in soil improvement and popularization of sustainable development concept in civil construction.

## 2. Unconventional Materials Used in Soil Improvement

### 2.1. Nanomaterials

Nanomaterial originates from nanotechnology and refers to the ultrafine materials with nanoscale (10^−9^ m) particle size. Nanomaterial has a very large specific surface area and high surface activity, resulting in many unique engineering properties different from those of traditional materials [9]. Currently, there are four types of nanomaterials commonly used for soil improvement: colloidal silica, bentonite, laponite and carbon nanotube.

#### 2.1.1. Colloidal Silica

Colloidal silica (CS) is an aqueous dispersion with nanoscale silicon dioxide particles produced by silicate solution. It is a new kind of nanomaterial used for soil improvement. CS is biologically and chemically inert. The particle size of silicon dioxide is generally between 2 to 22 nm [9,10]. During the manufacturing process, CS solution is kept alkaline so that silica particles repel each other and the CS stays ionized. When electrolyte solution or acid is added, repulsive forces decrease gradually, and siloxane bonds start to develop. Therefore, gelation can be induced. The gelation process of CS will bond soil particles together and improve the soil mechanical behaviors remarkably.

**Figure 1:**
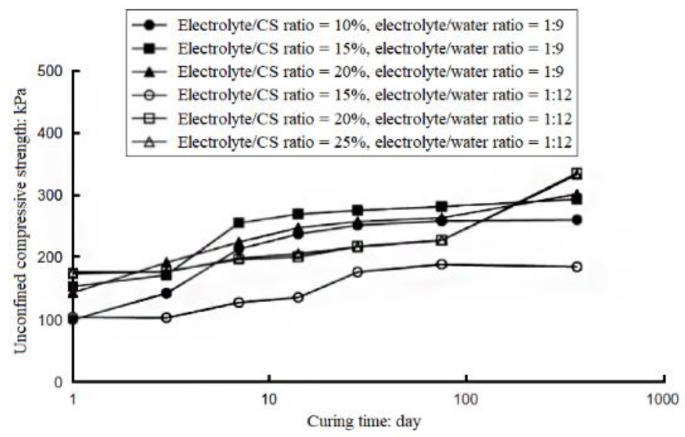
The relationship between UCS of colloidal-silica-treated soil and curing time [14].

Researchers found that the peak shear strength and stiffness of the CS-treated soil was greatly improved compared with the untreated one [11]. Moreover, a compaction test showed that the maximum dry bulk density of the CS-treated clay increases obviously compared with the pure clay sample, since CS particles can fill the voids of the sample. Moreover, the optimum moisture content also increases after CS treatment due to the moisture absorption of CS. The dense structure of the treated sample will help to improve the bearing capacity as a matter of course. The California bearing ratio (CBR) value of the clay sample treated with 0.5~1.0 wt.% CS increases by 38%~82% [12]. Moreover, the unconfined compressive strength (UCS) is obviously improved for sand sample after CS treatment, and the improvement degree is affected by CS concentration and curing time. CS-treated sand has an unconfined compressive strength that is increased with curing time, and the value is approximately doubled after a one-year curing period (Figure 1) [13,14,15]. In addition, CS can not only promote the static strength of CS-treated soils but can also improve dynamic resistance for sandy soil that is susceptible to liquefaction. Gallagher and Mitchell conducted an undrained cyclic triaxial test and found that CS significantly inhibits the development of axial strain during load cycles. After being treated with 10 wt.% of CS, the axial strain of loose sand never exceeded 2.5% during the whole 100 dynamic load cycles when cyclic stress ratio (CSR) was 0.27 [15]. Kodaka et al. found similar results in cyclic torsional shear tests. Under the same number of load cycles, the CSR needed to induce initial liquefaction for the CS-treated sand is about twice that needed for the untreated one [16]. By performing cyclic simple shear tests, Díaz-Rodríguez found that the number of cycles to liquefaction for the 10.8 wt.%-CS-treated sand is almost six times the untreated sand [17], indicating that the liquefaction resistance is obviously improved after CS treatment. Moreover, it has been investigated that the shear modulus of the treated sand was also improved in a resonant column test [18].

Since gelatinized CS is a network composed of particle chains [19,20], the pores in the treated soil are occupied by silicon particles and gel chains, which make the micropores smaller and impede the flow of water. Therefore, the hydraulic conductivity of CS-treated soil is expected to reduce. Actually, the hydraulic conductivity of the grouted sand is only 10^−8^ cm/s, which is a million times smaller than the pure sand [21]. Moreover, it has been investigated that hydraulic conductivity decreases exponentially with the increased concentration of CS particles on the condition that CS content goes beyond 7.4 wt.% [13]. Thus, the microstructure of the treated soil is greatly modified when CS is grouted into the soil mass. The pores are filled with CS colloids, which further bond the soil particles together. The cementation will undoubtedly improve the mechanical properties and decrease the permeability of the treated soil.

#### 2.1.2. Bentonite

Bentonite is a kind of highly plastic clay which mainly consists of montmorillonite. Bentonite has two basic properties: high water-absorption capability and thixotropy. Thixotropy is a kind of invertible sol phenomenon, which means the viscosity of bentonite varies with its status. It implies that the bentonite behaves like a liquid when stirred or shaken but sets back to gel when allowed to stand [22]. Up to now, the mechanism of thixotropy still lack a uniform interpretation. Thixotropy is a critical property for bentonite to be used as a grouting material. Once bentonite has gelled, soil particles are glued together after bentonite treatment.

**Figure 2:**
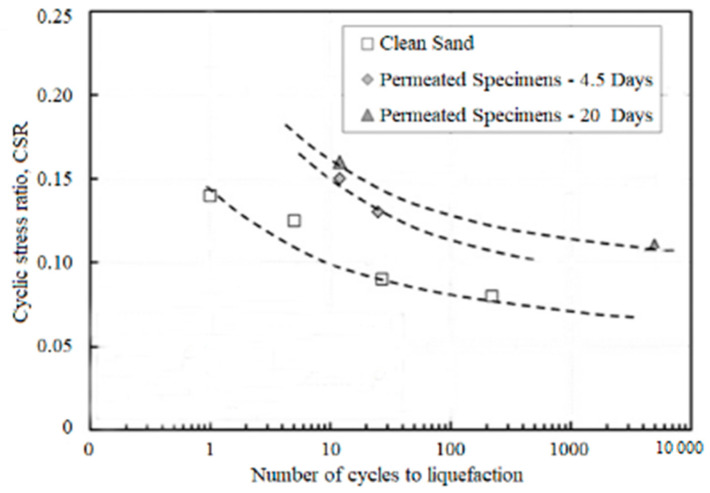
The relationship between cyclic stress ratio (CSR) and number of cycles to liquefaction for bentonite-treated samples with different curing time [25].

Bentonite has a great effect on resisting liquefaction of the reinforced soil. It is considered that the presence of bentonite could inhibit the increase of strain and delay excess pore pressure generation [23]. Undrained triaxial tests show that the magnitude of excess pore water pressure is reduced significantly for bentonite treated sand. Therefore, grouting bentonite suspension is regarded as an effective way to mitigate static liquefaction [24]. When dynamic load is applied, there is an order of magnitude increase in the number of cycles required for liquefaction for bentonite-treated sand at any given CSR compared to the pure sand. Moreover, the liquefaction resistance of the treated soil is increased with curing time to a large degree. For instance, the sample with a curing time of 20 days needs 20 cycles to induce liquefaction at CSR = 0.15, while the sample cured 4.5 days only needs 10 cycles to collapse, indicating a clear delay in pore pressure generation when curing time is increased (Figure 2) [25,26]. In addition, Witthoeft et al. implemented a bounding-surface-based constitutive model of the bentonite-treated sand in the finite difference software. The numerical simulation results indicate that the bentonite treatment could inhibit liquefaction effectively [27].

Similar to CS, the hydraulic conductivity is also greatly reduced for soil samples after bentonite treatment. Hwang H et al. conducted permeability tests on the sand samples treated with 5, 10 and 12 wt.% bentonite suspensions, and the hydraulic conductivities of the treated sand decreased by 4, 4.5 and 5 orders of magnitude, respectively. The lowest hydraulic conductivity of the treated sand is only approximately 8 × 10^−7^ cm/s [28]. When the bentonite content goes beyond 3 wt.%, bentonite could block all the sand voids and will decrease hydraulic conductivity more effectively. However, the bentonite portion filled in the pores of sand may experience washout when the external hydraulic gradient is large. The amount of washout is inversely proportional to the yield stress of the bentonite grout [29]. Remarkably, bentonite acts as the role of a binder when it is grouted in a soil mass. It will possess the pore space and minimize the permeation property of the treated soil. Of course, bentonite can help to strengthen the liquefaction resistance and thus it is regarded as an excellent grouting material.

#### 2.1.3. Laponite

**Figure 3:**
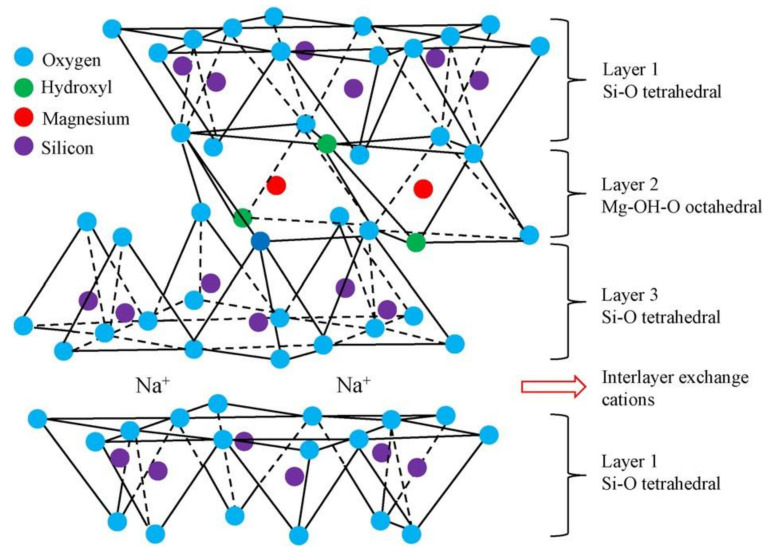
The microstructure of laponite [31].

Laponite is a synthetic quasi-montmorillonite, in which magnesium ions are partially replaced by lithium ions for the octahedral crystals of montmorillonite. The microstructure of laponite is depicted in Figure 3 [30,31]. The particle size of laponite is distinctly smaller than that of bentonite. When laponite concentration reaches about 3 wt.% in the suspension, the suspension will change from water-like liquid to solid gel over a few hours, which makes it possible for using laponite as a kind of grouting material [31].

**Figure 4:**
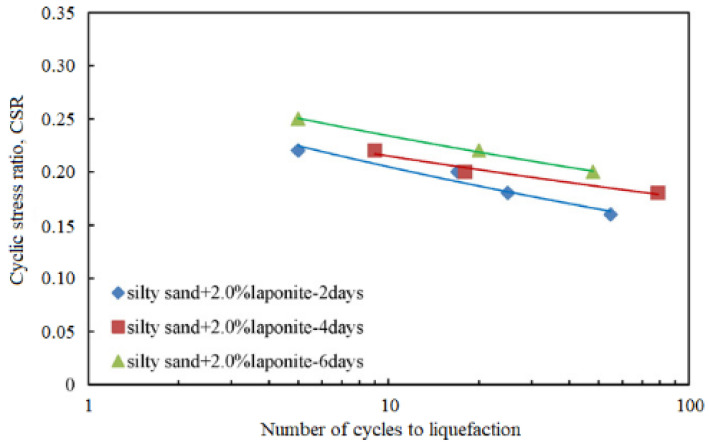
The relationship between CSR and number of cycles to liquefaction for laponite-treated samples with different curing time [31].

Laponite has an excellent performance in improving the dynamic properties of grouting soil. Based on its gelation property, laponite can be injected into sand to form a solid gel in the sand pores, which inhibits the flow of water in the pores of sand grains, delays the generation of pore pressure under dynamic load and thus improves the resistance of sand liquefaction. Under the same CSR, the anti-liquefaction ability of sand is significantly improved with the addition of laponite, and the number of load cycles to liquefaction increases by about two orders of magnitude [30]. As laponite delayed the formation and propagation of excess pore pressure, the response of pore pressure can be artificially divided into generation stage, propagation stage and dissipation stage [31]. In addition, the existence of laponite restricts the movement of soil particles and reduces the strain of the treated soil. The greater the laponite concentration, the more significant the improving effect of laponite. Moreover, curing time also presents a positive correlation with the strengthening effect for the laponite-treated soil, as shown in Figure 4. The increase of laponite content and the extension of curing time also leads to the increase of shear modulus and damping ratio, which further help to promote the soil stiffness [32,33]. However, the effects of laponite concentration and curing time on the liquefaction resistance are different. In the initial several loading cycles, laponite concentration is dominant factor controlling the progradation of liquefaction. After a few more loading cycles, the influence of curing time was stronger [33].

#### 2.1.4. Carbon Nanotube

Carbon nanotubes (CNTs) are tubes made of carbon with diameters measured in nanometers. As a kind of one-dimensional nanomaterial, CNT is made of graphene, with carbon atoms arranged in a hexagonal structure and rolled into a tube. This unique structure is responsible for the many unusual mechanical, chemical and electrical properties of CNTs. CNTs have two kinds of forms: single-walled carbon nanotube (SWCNT) and multi-walled carbon nanotube (MWCNT) [34]. The SWCNT typically has a diameter of 0.6–2 nm, with less defects and higher consistency than MWCNT. The diameter of MWCNT generally ranges from 2 nm to 100 nm, sometimes even reaching hundreds of nanometers, which leads to densely distributed pinhole-like defects on the tube wall. As CNTs can improve the compressive strength, flexural strength and fracture property when they are highly dispersed in cement matrix, carbon nanotubes are extensively used as a new cement additive in the field of civil engineering [35,36]. Recently, it was found that CNTs have a satisfactory performance when adopted as an additive for soil improvement.

Some scholars have added CNTs to the clayey soil and found that the addition of CNTs leads to an increase in the friction angle and a decrease in cohesion. Moreover, the unconfined compressive strength of 3% CNTs-treated soil was raised by about 120% compared with the untreated soil [9,37]. Besides, adding CNTs can increase the improving effect for the cement-reinforced soil. This is because the voids filled by CNTs are minimized and make the soil mass become denser. The shear strength of cement-reinforced soil in UCS test increases with the CNTs content when suitable dispersant is added. However, more CNTs would hinder the potential bond between cement and soil particles, and thus excessive CNTs will decrease stiffness and the strain at failure, which can induce a ductile behavior in the sample [38]. On the other hand, CNTs can not only play an important role in improving mechanical properties for a soil sample, but also can be used to absorb some organic material (polyaromatic hydrocarbons etc.) in the soil as they are highly absorbent [39].

However, unlike other nanomaterials mentioned above, there are concerns about CNTs’ impact on the soil environment. Li et al. found that CNTs can inhibit soil enzyme activity and reduce microorganism quantity [40,41]. The enzyme activity and microorganism quantity have a negative relation with the surface area or concentration of CNTs, and high concentration of CNTs may significantly alter and adversely affect soil microbial communities. While, in the opinion of Huang and Wang, the effect of CNTs is similar with C_60_ or nC_60_ and has little impact on the structure or function of soil microbial community and microbial processes [9,42], further research is needed on the potential application of CNTs in soil reinforcement.

### 2.2. Other Unconventional Materials

#### 2.2.1. Microbially Induced Calcite Precipitation

Microbially induced calcite precipitation (MICP) is a technique of inducing microorganisms to produce calcite (CaCO_3_) precipitation in an environment of urea and calcium ions. It is an interdisciplinary method of biology, chemistry and civil engineering [43]. Sporosarcina pasteurii is a kind of ureolytic bacteria that is commonly used in MICP technology, whose cells are usually 2 μm in diameter. The cells of Sporosarcina pasteurii do not aggregate, which leads to a high cell surface to volume ratio and ensures the efficiency of calcite precipitating [44]. For soil improvement, the culture medium which contains microbial nutrients and environmental conditions is injected into the pores of soil as grouting liquid. Then, the tiny pores in the soil will be filled with insoluble calcium carbonate precipitates induced by bacteria. Thus, the soil is reinforced. MICP slurry has good fluidity and low viscosity which required low magnitude of grouting pressure. Therefore, MICP is a kind of in-situ grouting method with low-energy demands and little-waste emission [45].

**Figure 5:**
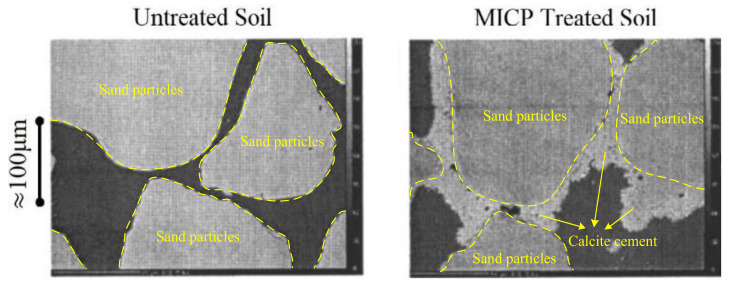
SEM graph of untreated and microbially induced calcite precipitation (MICP) treated soil [44].

**Figure 6:**
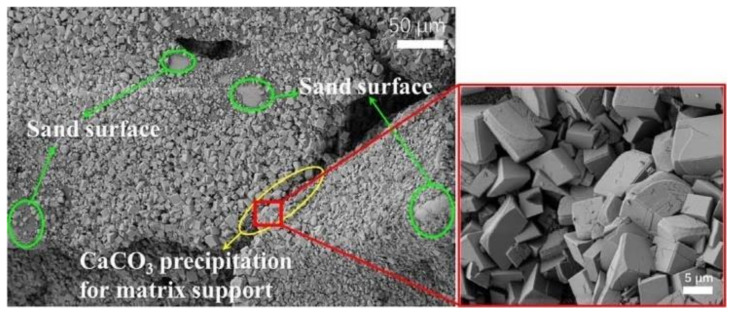
SEM graph of CaCO_3_ crystals formed in MICP-treated samples [47].

The applications of MICP technology in soil improvement has been discussed in detail in the past few decades. Ferris et al. investigated the cementation effect of MICP by preparing two groups of sand cores in Plexiglas tubes. One group of sand core was grouted with MICP solution, while the other was grouted with sterile water as a comparison [46]. After a period of bacterial culture, the treated sand core stuck together and became highly polished at the core holder surface. Therefore, it could not be removed from the holder except by cutting the Plexiglas away from the core. However, the untreated sand could be easily removed from the holder, since no cementation was formed during the same period of time. Although the MICP-treated sand core shows significant bonding between sand particles, it was still brittle and could be broken by sharp blows, and acid could also cause the core to disintegrate. Recently, scanning electron microscopy (SEM) images were used to directly observe and compare the particle characteristics for untreated and MICP-treated sand sample. As shown in Figure 5, untreated sand particles take on an irregular shape and are not bonded to each other. After treatment with MICP, the surfaces of sand particles are covered with a layer of light grey calcite cement. Although these granular calcite cement particles do not fill all the pores in the soil, many sand particles are still connected with each other by cementation effect [44]. Further observation shows that, under a lower magnitude of bio-cementation, most calcite cement crystals present cube-shaped particles and are distributed as grain coating, while only a few crystals are distributed as matrix supporting (Figure 6). When the magnitude of bio-cementation increases, the boundary between calcium carbonate and sand will be indistinguishable [47].

**Figure 7:**
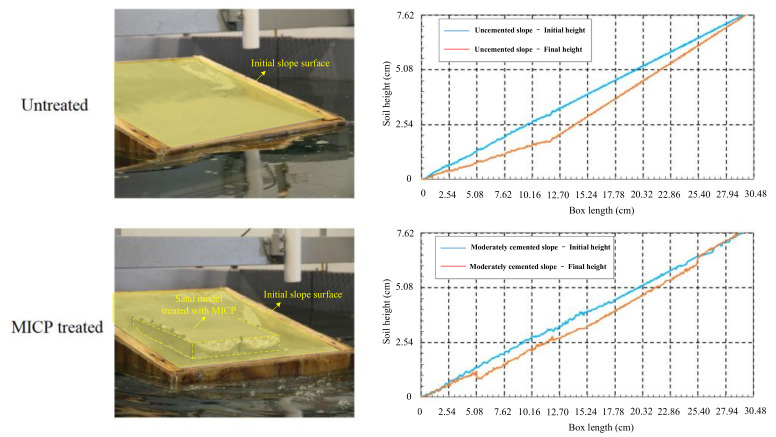
Erosion resistance of sandy slope treated by MICP (Modified from [53]).

MICP-treated soil would like to demonstrate improved static mechanical behavior with higher cohesion and unconfined compression strength, reduced permeability for preventing erosion and excellent liquefaction resistance when compared with a pure soil sample. First of all, the experiment results show that MICP can increase the cohesion of treated sand due to the cementation formed by the precipitation of calcium carbonate between the sand grains, but MICP had little effect on the friction angle [48]. Moreover, there is a strong linear relationship between the unconfined compressive strength of MICP-treated sand and the precipitation amount of calcium carbonate. The largest unconfined compressive strength of a treated sand column could reach as high as 800 kPa [49,50]. In the process of MICP treatment, repeated grouting is generally necessary to supply microorganism and nutrient solution to the soil sample to facilitate the cementation. It should be noted that the unconfined compressive strength has a positive correlation with the precipitation content of calcium carbonate as well as the treatment times [51]. Secondly, since calcium carbonate is insoluble in water, MICP technology can be used to reduce soil permeability, particularly to prevent erosion of sandy embankment. Even on the condition that hydraulic gradient of MICP-treated soil is 3~4 times higher than the untreated soil, the hydraulic conductivity of the treated soil decreases about 66% [52]. In a bench-model experiment, artificial wave erosion was carried out on MICP-treated and untreated slopes, and the morphology of the slope surface before and after wave action was recorded by laser scanner. The results show that the erosion of an MICP-treated slope is significantly reduced compared with the untreated slope (Figure 7) [53]. Furthermore, it is reported that there is a strong negative linear correlation between hydraulic conductivity and deposition content of calcium carbonate for the MICP-treated sand. When the content of precipitated calcium reached more than 1% weight percent, hydraulic conductivity of the treated sand is lower than 10^−4^ cm/s [50]. Of course, the average precipitation amount of calcium carbonate increases with the concentration of urea and calcium chloride in the solution, but the distribution of calcium carbonate in soil is not uniform [54].

**Figure 8:**
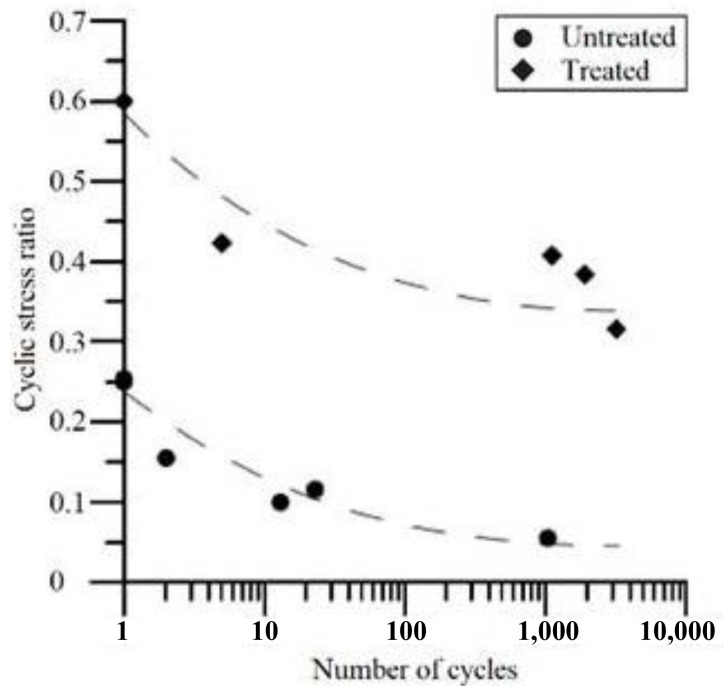
The relationship between cyclic stress ratio and number of cycles to liquefaction for sand samples treated by MICP [55].

More importantly, a series of model tests confirmed that MICP has an excellent effect on restricting soil deformation and thus improving the liquefaction resistance of sand, as shown in Figure 8. The CSR of both untreated loose sand and MICP-treated sand are plotted against the number of cycles needed to induce liquefaction. It is obvious that the MICP-treated sand needs a significantly larger number of cycles to liquefaction compared with untreated sand on the condition that the CSR remains the same, indicating that the liquefaction resistance is improved by MICP treatment [55]. Moreover, the acceleration and pore pressure response of pure sand and treated sand are compared in a centrifuge model test. The excess pore pressure ratio and acceleration in MICP-treated sample are significantly reduced compared with pure sand sample [56]. In addition, the vertical strain of the treated sand significantly decreased by more than 50% compared with the untreated sand, and the loose sand is just as likely to be densified after MICP treatment. Furthermore, the shaking table model test shows that the effect of MICP treatment in liquefaction resistance is stronger than gravel pile treatment. After the action of 0.5 g seismic waves, gravel-pile-treated model had 6 mm of water accumulated with 8.2 mm of settlement, while MICP treated model had no water accumulated w only 1.2 mm of settlement [45].

#### 2.2.2. Recycled Tire

With the rapid development of society, the amount of waste tires keeps increasing. The disposal of these wastes presents a serious challenge for environment protection. Recently, a possible solution was put forward by reusing waste tires in soil reinforcement, since tire rubbers can improve the shear strength, toughness and ductility of soil. Waste tires are mainly cut into tire chips or tire shreds when applied in soil reinforcement [57,58,59,60].

**Figure 9:**
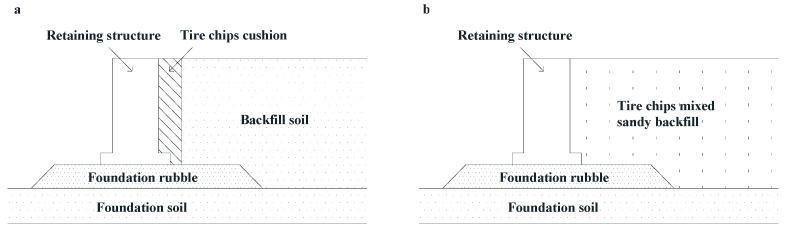
Tire chips used to protect retaining structure: (**a**) as a cushion or (**b**) as an additive to be mixed evenly in the backfill (Modified from [64,65]).

Tire chips have positive effect on the improving dynamic properties of treated soil. It has been investigated that when the volume of tire chips reaches 50% or more of the total volume, the maximum excess pore water pressure will always remain below the effective confining pressure, indicating the tire-chips-treated soil can resist liquefaction effectively [61]. The damping ratio of treated soil also increases with the tire chips content when the content is below 10%, but it tends to decrease when the chips content goes beyond 10% [62]. Moreover, the elasticity of rubbers makes it possible for tire chips to be used as protective cushion for structure through energy absorption during earthquakes [63]. Hemanta and Kohama verified the anti-seismic effectiveness of a tire chips cushion. They performed a shaking table test on the model shown in the Figure 9a. In the model, a buffer layer consisting of tire chips was set between the retaining wall and backfill soil. Test results show that the residual displacement increases quite slowly in a seismic event, and the earth pressure under dynamic load is much less compared with untreated model [64]. After this, Hazarika et al. performed another shaking table test by distributing the tire chips evenly in the backfill soil, as shown in Figure 9b. It can be concluded that tire-chip treatment can prevent the liquefaction of backfill soil [65]. Undoubtedly, the improvement in liquefaction resistance could be due to the low stiffness and buffering function of the tire chips. In the process of excess pore pressure development, tire chips can produce a certain compression which is similar to the situation of drainage, resisting pore pressure generation to a large extent [66]. Furthermore, it is worth noting that the size of the tire chips is an important factor that could remarkably affect the strengthening effect. It has been investigated that the shear strength of gravel-tire chips mixture increases with the ratio of median size of tire chips to that of gravel at a given relative density and confining pressure [67].

Tire shred can increase the ultimate bearing capacity of treated sand ground. In a plate bearing test, the bearing capacity increases almost linearly with the content or aspect ratio of tire shred. Tire shred with 60 mm in length and 20 mm in width can increase the bearing capacity of treated ground from 120 kPa to nearly 260 kPa at a tire shreds content level of 20%. More content and longer lengths of tire shreds can result in a higher bearing capacity of the treated ground, which may be due to the fact that the tire shred can provide tensile strength along the length of shred in the sand [68]. On the other hand, tire shred mass can maintain high levels of permeability, even in a compressed state. The hydraulic conductivity of tire shreds layer is about 1 cm/s, which is similar to gravel. Full-scale field trial shows that the tire shreds drainage layer performs satisfactorily [57]. However, shaking table test also shows that the maximum excess pore pressure ratio of treated sand increases with the content of tire shreds. It may due to the low permeability of tire shred itself, which leads to the accumulated pore water pressure [69].

Except for old tires, rubber particle recycled from old garden hoses, plastic shoes and other items can also increase the shear strength of the treated soil [70]. Specially, short rubber fibers can reduce the displacement rate and improve the ductility of sandy soil [71]. Moreover, adding waste broken glass powder or waste textile fibers also has positive effect on the strength and deformation performance of soil [72,73,74]. The recycling and reusing of these waste materials can effectively help to save resources and protect environments.

#### 2.2.3. Environmental Fibers

The application of fiber materials for soil improvement has lasted for a few decades. However, a systematical study on environmental fibers has been carried out only recently [10,75]. According to the formation cause of fiber materials, fibers in soil reinforcement can be divided into natural fibers and synthetic ones [76].

Natural fiber is low-cost and non-polluting. Since formal studies on natural fibers used in soil reinforcement have lasted for several decades, many research achievements on the static properties of the fiber-treated soil have been reported. Currently, common natural fibers that are studied are coconut fiber, sisal, palm fibers, jute, flax, barley straw, bamboo, cane, etc. [1]. These fibers can reduce the resilient strain and increase the shear strength of the composite soil. For instance, the resilient strain of the sample reinforced with 0.75% weight content coconut fiber is only about 0.3%, which is less than 0.4% resilient strain of the original sample [77]. Palm fibers with 30 mm length and 0.5% weight content increase the friction angle of soil from 30° to 36° [78]. Meanwhile, fibers can connect soil particles and aggregate into a continuous matrix. This makes the treated soil present a certain ductility before and after failure. The soil treated with straw fibers presents a large number of cracks in compressive test. However, the pure soil sample is brittle and collapses all of a sudden with only one crack [79].

Recently, some researchers also focused on the dynamics properties of the fiber-treated soil. Kirar B et al. reported that the addition of coconut fiber could increase dynamic shear modulus and reduce damping ratio of the sand sample. Under the same shear strain, the effect of fibers on shear modulus and damping ratio increases with the fiber content. The contribution of coconut fiber to shear modulus is more significant at high shear strain [80]. Moreover, some scholars also studied the reinforcement effect of human hair fibers on sand. It has been confirmed that human hair fibers have a significant effect on the shear strength and shear modulus of dry sand. However, the shear strength parameters of the treated and untreated sand are similar for saturated sand sample [81,82]. Since natural fibers can be categorized into plant fiber and animal one, plant fiber is more commonly adopted in soil improvement. It can improve both static and dynamic properties of the reinforced soil. Natural fibers originate from nature and return to the natural world. Therefore, it is an ideal additive for soil improvement in the context of sustainable development.

Synthetic fiber is generally highly molecular and difficult to degrade. Glass fiber is a kind of representative synthetic fiber that is commonly adopted in soil reinforcement. It is light in weight and has good biological degradability. The main composition of glass fiber is silica, which is a rich renewable resource [83]. At present, short glass fibers are mostly focused as additives for soil improvement. The length of these short glass fibers are generally only several centimeters, and fibers are randomly distributed in soil. It is indicated that short glass fiber could improve the stiffness and ultimate strength of the sand sample in a triaxial test. The strengthening effect is up to both the aspect ratio and content of the fiber. The growth rate of ultimate strength is in direct proportion to the aspect ratio of fiber. In addition, ultimate strength also increases linearly with the fiber content on the condition that fiber content is below 2%. When the fiber content reaches 2% of the total weight, ultimate strength increases to an upper limit [84]. This may be due to the fact that higher fiber content decreases the density of the soil and offsets the improving effect [85]. On the other hand, the water content of the treated soil could be slightly decreased due to the drainage effect of short glass fiber. This could help to reduce the effect of a freeze-thaw cycle on the soil. Relevant tests confirmed that the attenuation of elastic modulus, cohesion and internal friction angle induced by a freeze-thaw cycle can be inhibited by the treatment of short glass fibers [86]. Moreover, short glass fiber could also increase the shear modulus and damping ratio of the treated soil [87].

Except for being used alone, short glass fiber is more commonly applied as an additive for cement-reinforced soil. The addition of short glass fiber in cement-reinforced sand can enhance the strength and reduce the stiffness of the soil, which may be due to the increase in interparticle friction and bonding [83]. The failure mode of the cement-reinforced sample changes from brittle to ductile after short glass fiber treatment, and this effect is more significant at higher aspect ratio of the fiber [2,88]. Of course, there is also long glass fiber for directional improvement. However, the shape of long glass fiber is similar to a traditional geogrid or geotextile. Therefore, the strengthening mechanism of long glass fiber is similar to that of reinforced earth. Direct shear test showed that cohesive force and internal friction angle of the treated soil increased obviously with the number of glass fiber layers [89].

## 3. Evaluation of Different Unconventional Materials

### 3.1. Reinforcement Mechanism

The unconventional materials included in this paper can be divided into cementing materials and granular ones. Cementing material includes colloidal silica, bentonite, laponite and MICP, while granular material mainly contains recycled material and environmental fibers. Carbon nanotube is not summarized in the following because of its controversial effect on soil improvement.

**Figure 10:**
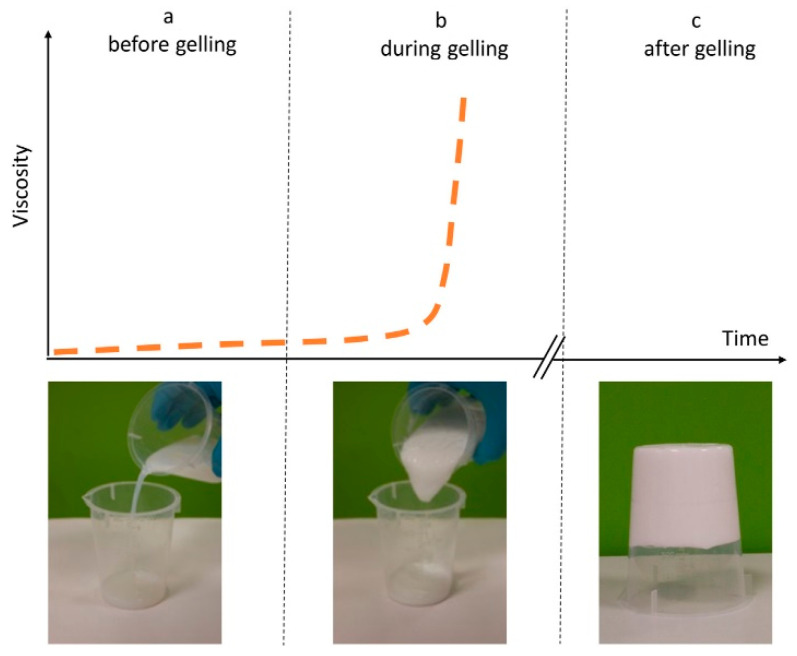
Gelation process of colloidal silica [11].

**Figure 11:**
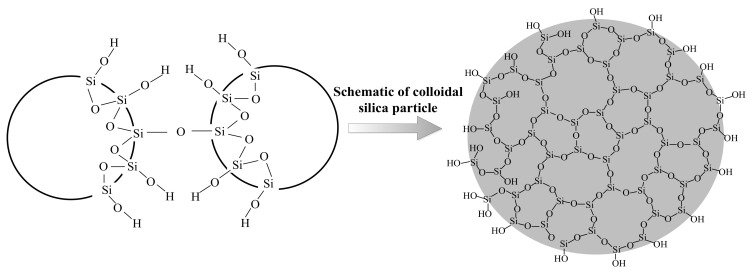
Formation of siloxane bonds and schematic of colloidal silica particles (Modified from [90]).

The principle of cementing material is bonding loose soil particles together through the cementation of additive. Although both nanomaterials and MICP belong to cementing materials, their reinforcement mechanisms are slightly different. Nanomaterials rely on gelling properties to strengthen the soil structure and improve mechanical properties. When nanomaterials are grouted, they can maintain a fluidity that is similar to pure water in the initial period. After a period of time, the slurry begins to gel and its viscosity can increase rapidly in a short time. Therefore, soil particles will be glued by the gel, and mechanical properties are improved. Take CS as an example, the viscosity of CS slurry increases slowly in the incipient stage, but it rises dramatically after a critical time node, as shown in Figure 10 [11]. The gelation of CS originates from the bonding of H_4_SiO_4_ molecules through the siloxane (Si-O-Si), as shown in Figure 11 [90]. Moreover, the gelation process can be motivated and promoted by adding electrolyte (i.e., NaCl, CaCl_2_ et al.) [91,92]. However, the microstructure of laponite after gelation presents elongated cells through Cryo-SEM observation, which is different from CS [93]. The longest dimension of the cell is about 20~50 μm, while the size of the laponite particles is only 25–50 nm. In addition, it is believed that laponite fines can also be attracted to the soil grains due to the charge attraction and that they form bonding or bridging at the particle contacts. Therefore, the laponite particle can make the soil particles bond more closely and have better effect than CS in soil improvement [94]. The gelation of bentonite reinforcement relies on its thixotropy. The initial viscosity of bentonite is relatively high and needs the addition of sodium pyrophosphate to improve its fluidity [95]. Moreover, the improvement of fluidity by sodium pyrophosphate is temporary. The viscosity of bentonite will increase with time when the slurry is left standing because of the thixotropy. In addition, thixotropy enables bentonite to recover the solid-like response and mechanical properties with time after the effect of dynamic load [96].

**Figure 12:**
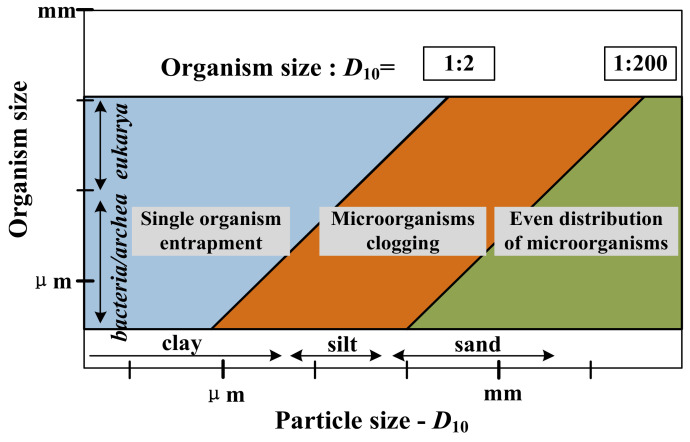
The relationship between microorganism size and soil particle size for even distribution (Modified from [101]).

MICP treatment relies on the biochemical reactions to induce calcium carbonate deposits rather than gelation to bond soil particles. The slurry of MICP is mainly produced from integrative action of ureolytic bacteria, nutrient solution and calcium ion solution. The biochemistry reaction in the grouted soil is shown in Equations (1)–(3). Urea in soil and nutrient solution are decomposed into NH_4_^+^ and CO_3_^2−^ through the effect of ureolytic bacteria, which increases the pH of the environment. Under this alkaline condition, carbonate ions combine with calcium ions to form calcium carbonate deposits, padding the pore in the soil mass and improving mechanical properties. In addition, calcium ions can also deposit and attach on the cell surface of microorganisms due to the opposite charge attracting effect, as shown in Equations (4) and (5). During the deposition, amorphous CaCO_3_ is first converted into vaterite and then into calcite, which is the most stable and least soluble of the known CaCO_3_ forms [97,98,99]. It should be noted that the even distribution of microorganisms is the primary factor controlling the reinforcement effect of MICP. Due to the Stokes’ drag law and interaction with mineral surface, the diffusion rate of bacteria in the porous soil network is lower than in slurry. Figure 12 divides the range of sizes for soil and microbial, where the soil particle size takes *D*_10_ as the indicator. When the sizes of soil particle and microbial locate on the left side of the parallelogram, microbial size is so large that single organism can be entrapped. When the particle sizes lie within the parallelogram, microorganism clogging may take place, and there will not be an even distribution in the soil mass. Even distribution of microorganisms can present only on the condition that the microbial size is small enough compared with soil particle size. Under this condition, the particle sizes locate on the right side of the parallelogram, and microorganisms are less likely to cause flow obstruction [100,101]. In addition, it seems possible to induce the native bacteria to produce more uniform calcium carbonate precipitation as the soil contains certain ureolytic bacteria [98].

(1) $$CO{\left(NH_{2}\right)}_{2}+2H_{2}O\to 2{\mathrm{NH}}_{4}^{+}{+\mathrm{CO}}_{3}^{2-}$$

(2) $${\mathrm{CO}}_{3}^{2-}{+H}_{2}O⇌{\mathrm{HCO}}_{3}^{-}{+\mathrm{OH}}^{-}$$

(3) $${\mathrm{Ca}}^{2+}{+\mathrm{HCO}}_{3}^{-}{+\mathrm{OH}}^{-}\to {\mathrm{CaCO}}_{3}↓{+H}_{2}O$$

(4) $${\mathrm{Ca}}^{2+}+\mathrm{Cell}\to {\mathrm{Cell-Ca}}^{2+}$$

(5) $${\mathrm{Cell-Ca}}^{2+}{+\mathrm{CO}}_{3}^{2-}\to {\mathrm{Cell-CaCO}}_{3}↓$$

The reinforcement mechanisms of granular materials are entirely different from those of cementing materials. At present, there is no clear mechanical model for the reinforcement mechanism of recycled materials like tire rubber. However, it is the difference in the stiffness of rubber and soil particle that results in the reinforcement. The stiffness of tire fragments is lower than that of soil particles. When the treated soil suffers external load, tire rubber in the soil can produce a certain compressive deformation. Therefore, the layer-placed tire chips could act as a buffer against the impact of the load. For the tire-shreds-treated soil, the compression deformation of tire shreds produce an effect similar to drainage, reducing and inhibiting the generation of excess pore pressure in the soil [66].

**Figure 13:**
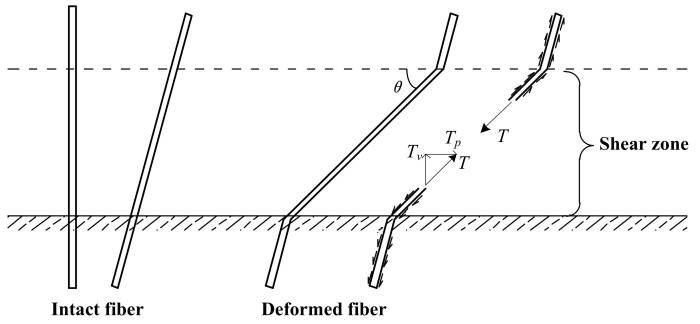
Long fiber reinforcement principle (Modified from [75]).

The reinforcement principle of environmental fibers differs from long fibers to short ones. The soil composite reinforced by long fibers has a total strength that consists of pure soil sample and reinforced fibers [102]. The ideal ultimate equilibrium reinforcement mechanism for one long fiber can be seen expressed in Figure 13, which is modeled on the assumption that the fiber could not be pulled out from the shear plane. Whether the fibers are perpendicular or oriented at any angle to the shear plane initially, the tension in the fiber can always be divided into the vertical and parallel components to the shear plane when the angles of fiber are known. The vertical component *T_v_* increases the compressive stress on the shear surface, improving frictional resistance in the sample. Meanwhile, the parallel component *T_p_* directly resists the shear force. On the condition of multiple fibers, the fiber content can be expressed by the ratio of the fiber cross-sectional area to the total shear surface. Therefore, the shear strength increasement can be calculated as the product of tension of one long fiber and the total fibers area in the shear zone [75]. As for short fibers, some existing studies have shown that short polypropylene fibers can make the soil more compacted by filling the soil pores and forming an interlock structure, which not only improves the static strength but also strengthens liquefaction resistance [103,104]. However, whether this principle can be applied to fiberglass remains to be studied, as the short polypropylene fibers are flexible material while glass fibers are rigid. The uncertainty of short fiber distribution makes it difficult to establish an accurate mathematical model for the reinforcement mechanism.

### 3.2. Reinforcement Effects and Characteristics

Different reinforcement mechanisms of these four kinds of unconventional materials lead to variations of reinforcement effects and characteristics. Among nanomaterials, the effects of CS are relatively more comprehensive. Not only can CS improve the static and dynamic properties of the treated sample, but also it can decrease the permeability of the soil. Bentonite is a kind of natural nanomaterials. It can mainly decrease the hydraulic conductivity and mitigate liquefaction when applied in soil improvement. The anti-seepage effect of bentonite is nearly equivalent to CS, both materials could decrease the hydraulic conductivity of the treated soil to only 10^−7^~10^−8^ cm/s [16,28]. Because of thixotropic characteristics of bentonite, the reinforced soil can recover a certain strength after suffering dynamic load. Therefore, bentonite has a much better capacity to withstand the shock of earthquakes and aftershocks compared with CS [96]. Laponite is a new kind of nanomaterial produced from bentonite and applied for soil improvement in recent years. Previous studies indicate that laponite has a better performance on reducing liquefaction risk than CS and bentonite. It is reported that liquefaction resistance of the sample treated with 3 wt.% laponite is similar to the sample treated with 10 wt.% CS. Also, the soil treated with 1 wt.% laponite behaved similarly to the sample treated with 3 wt.% bentonite [30]. In addition, nanomaterials have the common advantage that their viscosity is similar to that of pure water in the grouting process, which ensures a more even diffusion of slurry than traditional grouting materials. The effect of MICP technology in soil improvement is similar to that of CS. MICP can also increase the static strength, decrease the hydraulic conductivity and inhibit liquefication of the treated soil. However, the anti-seepage effect of MICP technology is slightly worse than CS and bentonite. It could only decrease the hydraulic conductivity of soil to about 10^−4^ cm/s [52]. In this respect, the principal effect of MICP is the resistance to water erosion since calcium carbonate deposits dissolve very poorly in water. However, the deposits particles can be corroded by acid, and the treated soil is more vulnerable to brittle failure [53].

Reuse of waste tire rubbers in geotechnical engineering helps to save resources effectively. As a kind of granular material used in soil improvement, the particularity of tire rubber lies in its compressible deformation. This characteristic makes tire rubbers absorb dynamic shock and inhibit liquefaction of sandy soil. The effect is especially significant when tire chips or shreds are put in layers in the soil [105]. Moreover, relying on its adsorption of volatile organic compounds like toluene, dichloromethane and trichloroethylene, tire rubbers can be used as leachate collecting layer in landfill [106]. However, tire rubber may increase the hydraulic conductivity of the soil sample, although the rubber itself is impermeable to water. The hydraulic conductivity of tire chips layer is about 1 cm/s, which is similar to gravel [57]. Fiber materials mainly increase the static strength and ductility of the soil and have little effect on permeability or dynamic properties. The reinforcement effect of fibers varies slightly with fiber length and texture. The reinforcement of directional reinforced long fibers, which are similar to geotextile or geogrid, is affect by the arrangement of fiber layers. However, the reinforcement effect of the samples treated with randomly distributed short fibers is reliant on the fiber content. Both natural fibers and synthetic ones have their own advantages and disadvantages. Natural fiber is low-cost and non-polluting, but it could not be adopted in long-term reinforcement as it could rot in soil. Synthetic glass fiber is a rich renewable resource and its durability is better than natural fiber, but it is brittle and has poor wear resistance.

The characteristics of each unconventional material are summarized and listed in Table 1.

## 4. Price/Performance Ratio

The price/performance ratio is a dominant factor for determining whether the unconventional materials can be widely used in practice. The market price of each kind of unconventional material is adopted to evaluate its price/performance ratio. For comparison, it is assumed that the porosity of soil to be treated is about 42%. On this condition, the treatment costs of these unconventional materials are calculated per cubic meter of treated soil.

It has been investigated that a 30 wt.% concentration of CS costs $0.88 per kg. However, researches show that a 5 wt.% concentration of CS is sufficient to mitigate liquefaction for loose sand [15]. Therefore, CS with 30 wt.% concentration can be diluted to 5 wt.% with de-aired water before use. After dilution, the 5 wt.% concentration CS only costs $0.15 per kg. Accordingly, the cost of CS is about $62 per cubic meter of treated sand. Bentonite with montmorillonite content beyond 95% costs $4.4 per kg. Since 5 wt.% concentration of bentonite can also effectively inhibit liquefaction [23], the cost per cubic meter of treated soil is about $97. Obviously, the cost of bentonite treatment is slightly higher than CS. Laponite RD is one of the most widely used laponite products and costs $24 per kg. Since laponite particles are finer than CS and bentonite, only a 3 wt.% concentration of laponite can achieve the equivalent effect for soil reinforcement. At this point in time, laponite treatment costs $302 per cubic meter soil, which is significantly more expensive than CS and bentonite.

The MICP technology is still in the experimental and exploratory stage. The reinforcing effect is related to multiple factors, such as bacteria concentration, amount of nutrient solution, calcium sources and so on. Therefore, its cost is difficult to estimate. Ideally, the bacterial strain for MICP treatment need be purchased only once, and the cost of freeze-dried powder Sporosarcina pasteurii is about $113 per tube. Then the bacteria can be put into culture solution for reproduction. By reproduction of the bacteria, MICP can be multiplied to be adopted as a grouting material. In other words, only the cost of nutrient solution is needed if the bacterial strain has already been purchased initially. Therefore, MICP treatment is expected to have the lowest cost when the relevant technology matures.

The recycled material can be laid out as a cushion or mixed with soil as an additive. When it is used as a cushion, a thickness of 0.3 m is sufficient to protect the structure [64,106]. Thus, rubber with a volume of 0.3 m^3^ is needed per square meter of the cushion layer. Given the fact that the volume of rubber in a tire is about 6500~8000 cm^3^ and the cost of a used tire is about $4~$7, the cost of the recycled material is about $150~$323 per square meter when it is used as cushion layers. When mixed with soil, shaking table tests show 10% weight content of tire shreds can effectively increase the damping ratio of soil [69]. Since the densities of soil and tire are 1.7 g/cm^3^ and 1.07 g/cm^3^ respectively, the cost of tire is about $75~$161 per cubic meter soil.

The cost of environmental fibers depends on the fiber type. Since the nature fibers are mostly local materials, their costs are difficult to estimate. For synthetic fibers, long glass fiber with 1 m width and 30 m length cost about $8.5 per piece. However, the total treatment cost for long glass fiber varies greatly according to fiber arrangement. The short glass fiber costs only about $0.5 per kg. It has been verified that the soil reaches the upper limit value of ultimate strength when 2% weight content of short glass fiber is added [84]. Based on this, the cost of short glass fiber is about $17 per cubic meter soil with soil density of 1.7 g/cm^3^.

The price/performance ratios are summarized in Table 2. The costs of several traditional materials are listed here for comparison [9]. It is clear that the unconventional materials have higher costs compared with cement, but most of them are much cheaper than chemical grouting materials, i.e., sodium silicate, acrylate grout and epoxy grout. Since cement has much greater particles and significantly higher viscosity than nanomaterials and MICP, it cannot permeate through voids of soil mass by itself and just has to rely on grouting pressure. Thus, cement treatment will undoubtedly cause intense disturbance to surroundings. Moreover, the manufacturing process of cement is energy-intensive, which belies the original intention of sustainable development. Although chemical grouting materials only have slight disturbance to surroundings, they may cause underground water pollution to a large extent. Therefore, unconventional materials have more excellent properties and are more environmentally friendly compared with traditional materials. Moreover, their cost is perfectly acceptable.

Among unconventional materials, nanomaterials reinforcement is the closest to application. Actually, colloidal silica (CS) has already been adopted to strengthen the ground under the runway and around common ducts of Fukuoka International Airport. CS is the cheapest nanomaterial in soil improvement and has the broadest market application prospects. Laponite is the most expensive of investigated unconventional materials, but its properties are superior to those of other nanomaterials in mitigating liquefaction. Since bacteria can reproduce in culture solution, only culture cost is needed once the bacteria strain is purchased initially. Thus, MICP tends to have the lowest cost in the future. Recycled tire treatment is slightly more expensive than CS treatment: its price/performance ratio is similar to bentonite. Short glass fiber is the cheapest among the investigated unconventional materials (MICP excluded), whose treatment cost is just slightly higher than that of cement. With booming market development, unconventional materials, especially nanomaterials, will likely have a much lower price in the near future, which will prompt their widespread application in the context of sustainable development.

## 5. Conclusions

In the past few decades, research was universally performed on unconventional materials for their application in civil engineering with the concept of sustainable development. Under this trend, various kinds of unconventional materials were adopted as additives for soil improvement. The unconventional materials adopted to reinforce soil mass are grouped into cementing materials and granular ones in this paper. The mechanical property, reinforcement effect, mechanism and price/performance ratio of four kinds of typical unconventional materials are discussed in detail to clarify their unique properties and applications in soil improvement. Major conclusions can be drawn by reviewing the research on these unconventional materials:

(1) Three kinds of cementing nanomaterials (i.e., colloidal silica, bentonite and laponite) have a more satisfactory reinforcement effect and higher price/performance ratio compared with traditional grouting materials and other unconventional materials. Therefore, nanomaterials have a broad prospect of development and application. Actually, colloidal silica has already been adopted for ground improvement under the runway and around common ducts of Fukuoka International Airport.
(2) Colloidal silica is the most widely used nanomaterial for soil improvement up to now due to its comprehensive improvement, satisfactory effect and low price/performance ratio. However, laponite has attracted more attention of researchers in recent years since it has better performance in mitigating liquefaction. It has been shown that soils treated with 3% laponite have a similar liquefaction resistance to soils treated with 10% colloidal silica.
(3) Microbially induced calcite precipitation (MICP) is an environmental-friendly material with remoldability and spontaneity. The calcite precipitation induced by microorganisms has a comprehensive effect on improving shear strength, adjusting permeability, mitigating liquefaction, purifying the environment, restoring metal contamination and so on. However, the selection of bacteria should be frequently adjusted within the engineering environment. The by-products, such as ammonium ion, nitrite, nitrous oxide, etc., should be cleaned in case of pollution. Nevertheless, MICP treatment will have the lowest cost if the relevant techniques mature in the future.
(4) Granular materials have properties and reinforcement mechanisms that are quite different from cementing materials. They do not initially have low viscosity and ideal fluidity similar to pure water, so granular materials cannot be adopted for ground improvement in developed areas. On the other hand, granular materials have an excellent performance in reinforcing reconstituted soil, and tire chips and shreds may act as a cushion to protect soil structures.
(5) Despite the satisfactory effect and high price/performance ratio of nanomaterials for soil improvement, there is also a growing concern that nanomaterials may have some unwanted or negative interactions with different organisms and the environment. Therefore, it is urgent that more environmental implication studies be carried out to verify the feasibility of nanomaterials adopted as additives for soil improvement.

## Figures and Tables

**Table 1:** Comparison of properties for the investigated unconventional materials.

Materials	Physical Properties		Mechanical Properties		Other Features
	State	Grain Size	Reinforcement Effect	Durability	
Colloidal silica	Slurry	7~100 nm [10]	Shear strength improvement; Resistance to deformation; Liquefaction mitigation; Seepage control	Great durability, strength and stiffness increased with curing time [14]	Electrolyte (NaCl or CaCl_2_) needed to induce gelation
Laponite	Slurry	1 nm in thickness, 25 nm in diameter [31]	Liquefaction mitigation		No additive needed to induce gelation; pyrophosphate or polyethylene oxide needed to inhibit gelation [107]
Bentonite	Slurry	within 1 μm [94,108]	Liquefaction mitigation; seepage control		Sodium pyrophosphate needed to decrease initial viscosity; Thixotropy; a certain degree of reinforcement recovery after earthquake
Carbon nanotube	granular	2~20 nm in diameter	Shear strength improvement; unconfined compressive strength improvement	Great durability	/
MICP	Slurry	20~120 μm for particles formed by microbial flocculation [45]	Shear strength improvement; Resistance to deformation; Liquefaction mitigation; Seepage control	Great durability	Multiple grouting required to supplement microorganisms, nutrients and calcium sources
Recycled material	Chip or shred	3~76 mm for tire chips; 4 ~19 mm for tire shreds [57,60]	Shear strength improvement; Resistance to deformation; Liquefaction mitigation; Ductility improvement	Great durability	Adsorption of volatile organic compounds
Natural fiber	Bundle	10~4000 μm in diameter; 5~500 mm in length [1]	Shear strength improvement; Resistance to deformation; Ductility improvement	Poor durability, biodegradable; putrescible	/
Synthetic fiber	Bundle or shred	For short fiber, about 0.3 mm in diameter and 6.4~25.4 mm in length; For long fiber, 0.12~1.1 mm in width and 35 mm in length [84,88]	Shear strength improvement; Resistance to deformation; Ductility improvement	General durability; Not abrasion resistant	Additive for other-material-reinforced soil to improve ductility

**Table 2:** Comparison of cost performance of different materials.

Categories	Materials	Content	The Unit Price	Cost (Per m^3^)
Conventional materials	Cement	5% weight content of soil mass	$0.1 per kg	$10 [9]
	Sodium silicate	/	/	$180 [9]
	Acrylate grout	/	/	$325 [9]
	Epoxy grout	/	/	$500 [9]
Unconventional materials	Colloidal silica	5% weight content of solution	$0.88 per kg (CS concentration 30%)	$62
	Bentonite	5% weight content of solution	$4.4 per kg (montmorillonite content beyond 95%)	$9
	Laponite RD	3% weight content of solution	$24 per kg	$302
	MICP	/	$113 per tube (freeze-dried powder Sporosarcina pasteurii)	/
	Old tires	10% weight content when mixed with soil	$4~7 per tire (volume of rubber of each tire is about 6500~8000 cm^3^, density is 1.07 g/cm^3^)	$75~161
	Long glass fibers	/	$8.5 per piece (1 m width and 30 m long)	/
	Short glass fiber	2% weight content of soil	$0.5 per kg	$17
